# A reverse genetics cell-based evaluation of genes linked to healthy human tissue age

**DOI:** 10.1096/fj.201600296RRR

**Published:** 2016-10-03

**Authors:** Hannah Crossland, Philip J. Atherton, Anna Strömberg, Thomas Gustafsson, James A. Timmons

**Affiliations:** * Division of Genetics and Molecular Medicine, King’s College London, Guy’s Hospital, London, United Kingdom; and; † School of Medicine, University of Nottingham, Royal Derby Hospital, Derby, United Kingdom; and; ‡ Department of Laboratory Medicine, Clinical Physiology, Karolinska University Hospital, Stockholm, Sweden

**Keywords:** epigenetic, neuromuscular, rapamycin, siRNA

## Abstract

We recently developed a binary (*i.e.,* young *vs.* old) classifier using human muscle RNA profiles that accurately distinguished the age of multiple tissue types. Pathway analysis did not reveal regulators of these 150 genes, so we used reverse genetics and pharmacologic methods to explore regulation of gene expression. Using small interfering RNA, well-studied age-related factors (*i.e.,* rapamycin, resveratrol, TNF-α, and staurosporine), quantitative real-time PCR and clustering analysis, we studied gene–gene interactions in human skeletal muscle and renal epithelial cells. Individual knockdown of 10 different age genes yielded a consistent pattern of gene expression in muscle and renal cells, similar to *in vivo.* Potential epigenetic interactions included *HIST1H3E* knockdown, leading to decreased *PHF19* and *PCDH9,* and increased *ICAM5* in muscle and renal cells, while *ICAM5* knockdown reduced *HIST1H3E* expression. Resveratrol, staurosporine, and TNF-α significantly regulated the *in vivo* aging genes, while only rapamycin perturbed the healthy-age gene expression signature in a manner consistent with *in vivo.* *In vitro* coordination of gene expression for this *in vivo* tissue age signature indicates a degree of direct coordination, and the observed link with mTOR activity suggests a direct link between a robust biomarker of healthy neuromuscular age and a major axis of life span in model systems.—Crossland, H., Atherton, P. J., Strömberg, A., Gustafsson, T., Timmons, J. A. A reverse genetics cell-based evaluation of genes linked to healthy human tissue age.

Chronological age is a major correlate for many diseases, including Alzheimer disease, cancer, and cardiovascular diseases. It has been hypothesized that differences in the rate of biologic aging represent an important reason why, along with key environmental factors, each human can develop age-correlated disease at different chronological ages (1). For this reason, it is important that robust biomarkers of biologic age are developed to facilitate a more personalized approach to health care. For example, such a biomarker could be applied to middle-aged subjects for the purpose of offering disease prevention advice. The analysis of gene expression changes with aging using microarray technology represents one important technology to realize this aim. Indeed, several meta-analyses studying different tissues and organisms have been used in an attempt identify common signatures of aging (2–6).

The majority of studies have used linear regression to build models that span <20 to >100 yr (6–8). However, when studying humans, this type of molecular profiling incorporates not only markers of age but also clinical disease and complex interactions with drug treatment that are currently impossible to deconvolute. Further, as a result of population stratification and changes in the genetic contribution to survival beyond 80 yr (9), these types of linear correlative approaches are problematic. We recently took a different approach: we used machine learning to distinguish muscle tissue obtained from healthy older subjects with functional capacity matched to younger subjects (*i.e.,* a binary classifier and not a linear correlative model). Using thousands of samples, we validated a 150-gene RNA tissue diagnostic able to accurately (>90% performance in independent samples) classify young from older human muscle, brain, and skin tissue (5). Gene expression changes with age were hypothesized to be adaptive, and hence regulation of the gene expression pattern is potentially beneficial to health. Notably, given that the signature was discovered in human muscle, exercise training does not regulate the healthy aging profile. Indeed, when comparing chronological age–matched cases and controls, this reproducible signature of tissue age was diagnostic for cognitive status in 2 independent cohorts as well as being related to renal function over a 12-yr follow-up period in older men belonging to a birth cohort and hence the same chronological age (10). In each study, the more the gene expression pattern matched the pattern observed in healthy older muscle tissue (5), the better the health status the older person had compared to someone with a similar chronological age but lower gene score.

Surprisingly, pathway and gene ontology analysis failed to identify any dominant pathway in the 150 genes (5). Individually, some of the 150 genes did show links to published age-related phenotypes, such as *LMNA,* a gene encoding a nuclear lamina protein that is linked to Hutchinson-Gilford progeria syndrome (11). However, for the majority of the genes, little was known about their biologic function, particularly in relation to aging. Nevertheless, our analysis in multiple human cohorts suggests that regulation of these genes may contribute to healthy aging, and investigation of how these genes are regulated or interact is therefore required to provide greater understanding into mechanisms of human aging.

The main aim of the present study was to determine whether interactions existed between individual members of the gene set, and in particular whether we could replicate the pattern of regulation *in vivo.* To achieve this aim, we used small interfering RNA (siRNA)-mediated gene knockdown and putative pro- (staurosporine and TNF-α) or anti- (resveratrol and rapamycin) age-related molecules. We found that the age classifier genes demonstrated coordinated changes *in vitro* that paralleled the up-regulation of gene changes with age *in vivo.* Further, we found evidence that rapamycin induced expression changes reflecting the same directional changes observed *in vivo* in healthy human muscle with age.

## MATERIALS AND METHODS

### Cell culture, siRNA, and drug treatments

Human primary skeletal muscle cells were established from muscle biopsy samples from healthy young adult men as previously described (12). This procedure was approved by the ethics committee of the Karolinska Institutet (Stockholm, Sweden). Isolated myoblasts (passages 4 and 5) were cultured on uncoated 12-well plates in DMEM/Nutrient Mixture F-12 (Thermo Fisher Scientific, Waltham, MA, USA) containing 20% (v/v) fetal bovine serum (Sigma-Aldrich, St. Louis, MO, USA), 4 mM l-glutamine, and 1% (v/v) antibiotic–antimycotic solution (Thermo Fisher Scientific). Cells were cultured at 37°C with 5% CO_2_. When cells were approximately 90% confluent, differentiation was induced by switching the medium to DMEM/F-12 containing 2% (v/v) horse serum (Sigma-Aldrich), 4 mM l-glutamine, and 1% (v/v) antibiotic–antimycotic solution (Thermo Fisher Scientific). Human primary renal epithelial cells were obtained from Promocell (c-12665; Heidelberg, Germany) and were cultured in Promocell growth medium. siRNA experiments were performed at passages 5 and 6. When cells were approximately 50% confluent, they were transfected (Lipofectamine RNAiMax) with 20 nM siRNA (same siRNAs as for the muscle cell experiments). After 48 h, cells were collected in Trizol (Thermo Fisher Scientific).

For each siRNA experiment, 2 separate siRNA sequences were used [On-TargetPlus from Dharmacon (Lafayette, CO, USA) or Silencer Select from Thermo Fisher Scientific], and 4-well replicates were used for each treatment. Three to 4 independent experiments were carried out for each siRNA treatment and cell type. For siRNA transfection of primary muscle cells, when myoblasts were approximately 100% confluent, they were switched to differentiation medium and transfected 48 h thereafter. Cells were transfected with 20 nM siRNA targeting *AIMP2, NPEPL1, PHF19, ICAM5, SLC38A10, HIST1H3E, LMNA, RUNX1, SHISA4,* and *CARM1* using Lipofectamine RNAiMAX transfection reagent (Thermo Fisher Scientific). Sequences of siRNA used were as follows (5′ to 3′): CAUAAUGCUGUCAACGCAA and CCAAUGCGCUGGACUUGAA for *AIMP2,* GGACAUGUCUGCUUAUUGC and GAAGGAGAUUUGCACAGGA for *CARM1,* GCGCAUCGCUGCAUUGUGA and GCAAUGAGAUGAACACCGA for *NPEPL1,* UGUGGUACCUGGACCGGAA and GGAGCUGAACUAGAACUAU for *SHISA4,* CCGGAGAUGGAUGAAUCUA and GUGACCAUCUACAGCUUCC for *ICAM5,* GGCAGAAACUAGAUGAUCA and GCUGAGCUGAGAAAUA for *RUNX1,* AGAAACAAGAGCCGGAGCA and AGCCGUCAGUGAAAACCAU for *SLC38A10,* CCAAAAAGCGCAAACUGGA and GAAGGAGGGUGACCUGAU for *LMNA,* GACUUGAUGUCCAAACUGA and GCCUCGUGACUUUCGAAGA for *PHF19,* and CCAGAAGUCUACCGAGCUU and GCGUGAAUUGUUUUGAGUA for *HIST1H3E*. Controls were performed with transfection reagent only, and each siRNA was tested for efficacy in initial experiments. Toxicity was checked in initial experiments to assess viability of the cells (annexin V/propidium iodine staining). Cells were collected in Trizol 48 h after transfection.

For drug treatment studies, experiments were performed on human primary muscle cells 6 d after switching to differentiation medium and 24 h after a medium change. Myotubes were treated for 24 h with the following chemicals: 100 nM rapamycin (Sigma-Aldrich), 50 μM resveratrol (Sigma-Aldrich), 10 ng/ml TNF-α (Sigma-Aldrich), and 10 nM staurosporine (Sigma-Aldrich). Bovine serum albumin [BSA; final concentration 0.0001% (w/v)] was used as a vehicle control for TNF-α, and DMSO [final concentration 0.03% (v/v)] was used as a vehicle control for rapamycin, resveratrol, and staurosporine. Cells were collected in Trizol, and each experiment was repeated over 3 passages (*n* = 4 well replicates for each experiment).

### Annexin V/propidium iodide staining of human renal epithelial cells

Human renal epithelial cells were cultured in 6-well plates and transfected with 20 nM siRNA at ∼50% confluence. After 48 h, the medium was removed, and cells were washed in warm PBS. Cells were incubated in a solution containing annexin V–FITC conjugate and propidium iodide using an annexin V–FITC apoptosis detection kit (Sigma-Aldrich). After 10 min incubation at room temperature, cells were fixed using ice-cold methanol:acetone, washed in PBS, mounted, and stained with DAPI using Fluoroshield mounting medium with DAPI.

### RNA extraction, cDNA synthesis, and real-time quantitative PCR

RNA was extracted using Trizol according to the manufacturer’s protocol. RNA was resuspended in 20 μl of RNase-free water and quantified using a NanoDrop (Thermo Fisher Scientific). RNA (500 ng) was reverse transcribed using the High Capacity cDNA synthesis kit (Thermo Fisher Scientific). cDNA was diluted 1:5, and real-time quantitative PCR (qPCR) was performed using 1 μl cDNA in triplicate with 10 μl master mix containing the following: SYBR Select Master Mix (Thermo Fisher Scientific) and primers designed to target the following genes: *AIMP2, NPEPL1, CALR, PHF19, ICAM5, SLC38A10, HIST1H3E, LMNA, RUNX1, SHISA4, CARM1, SIRT5, MBNL1, SKAP2, CFLAR, ALDH6A1, TGFBR3, CD36, RBMS3,* and *PCDH9*.

The following primer sequences were used (5′–3′): *AIMP2,* forward TGGGATTCACTTTAATTTGGAAG, reverse GTTCCCTTCGCCTTCGAT; *CARM1,* forward AACCACACCGACTTCAAGGA, reverse AAAAACGACAGGATCCCAGA; *NPEPL1,* forward GGGCAAAGGCATCGTCTA, reverse CCCGGCATGGTAGTCTTC; *SHISA4,* forward GGCCCCCAGTCTACAACC, reverse ATCTCCCAAGGTTGGCATC; *ICAM5,* forward GAGTCCTGATGTCACCCTCG, reverse CCGGGAAGCTGTAGATGGTC; *RUNX1,* forward CTCCCTGAACCACTCCACTG, reverse TGGGGATGGTTGGATCTG; *SLC38A10,* forward CGTCAGTGAAAACCATGAGC, reverse AGCTGACGTAGCCGAAAAAC; *LMNA,* forward CTGGTCACCCGCTCCTAC, reverse TGGCAGGTCCCAGATTACAT; *PHF19,* forward AAAGTTTTTGCTGGAAGATGCT, reverse TTGAATTTCATCCAGCGTGA; *HIST1H3E,* forward CTCGTAAATCCACAGGCGGT, reverse TAGCGATGGGGCTTCTTCAC; *CALR,* forward CTATGATAACTTTGGCGTGCTG, reverse ACTCCTCAGCGTATGCCTCA; *SIRT5,* forward GCTCGGCCAAGTTCAAGTATG, reverse CCTCTGAAGGTCGGAACACC; *MBNL1,* forward CTGCCCAATACCAGGTCAAC, reverse GGGGAAGTACAGCTTGAGGA; *SKAP2,* forward TCCCTAACATGCCCAACC, reverse TTTTCTCCTTTCAGTATATCTGCTACA; *CD36,* forward CCTCCTTGGCCTGATAGAAA, reverse GTTTGTGCTTGAGCCAGGTT; *PCDH9,* forward GCCCCTTACACACCAGACAG, reverse TTCATTCTCCTGAATGTGGAAA; *ALDH6A1,* forward CAGGTCTTGCTCCGCTATCAA, reverse GGCATGCTCAACCACCTGAAG; *RBMS3,* forward AGTTCCCTGCCTCGGAGATA, reverse CTGGTGCATAGGACTGCTTG; *CFLAR,* forward AAGTCCGCTTCCAGGCTTTC, reverse TCCAGTGGGGGAGTTCGTC; *TGFBR3,* forward CCAGCTACAGAGAGAGGTCAC, reverse CCACAGAACCCTCAGACACC; *ACTB,* forward GAGCACAGAGCCTCGCCTTT, reverse CATCATCCATGGTGAGCTGG.

Thermal cycling conditions were 2 min at 50°C, 10 min at 95°C, and 40 cycles of 15 s at 95°C and 1 min at 60°C. Samples were run on a Viia 7 qPCR machine (Thermo Fisher Scientific). For normalization, β-actin (*ACTB*) expression was measured (none of the treatments affected β-actin expression; data not shown). The Δ*C_t_* method (13) was used to calculate relative changes in target mRNA abundance.

### Statistical analyses

For siRNA experiments, 2-way ANOVA with Sidak’s multiple comparison test was used to test for the main effects of each siRNA treatment and the statistical significance of each gene between control and siRNA-treated groups, correcting for multiple testing. For drug treatment experiments, 2-way ANOVA was initially used to test whether each compound had an overall effect on mRNA expression. Our second aim with this analysis was to determine whether the changes in gene expression with each drug treatment reflected the directional patterns seen with aging *in vivo* (*i.e.,* whether genes up-regulated *in vivo* with aging were up-regulated by the drug treatment and *vice versa*). For those genes affected by the drug treatment, genes were assigned either 1 (if its direction of change matched the observed change with age *in vivo*) or 0 (if its direction of change was opposite to the observed change with age *in vivo*). Fisher’s exact test was used to determine the significance of the observed changes *vs.* the expected changes. Thus, a result of *P* < 0.05 indicated that the gene expression changes with each drug treatment were different from the directional changes *in vivo* with aging. Overall, Fisher’s exact test allowed us to assess consistency (*i.e.,* whether the drug RNA response was similar or distinct from the *in vivo* pattern of changes with muscle tissue aging).

Distance-based cluster analysis was used assess the relationship between gene expression responses across all experiments. The analysis was done in the R statistical environment (*https://cran.r-project.org*) using the library package Library(gplots) and Euclidean-based distance, with the setting Complete. Gene expression was calculated as a percentage of control, and row- and column-based clustering was implemented. This approach represents a robust method for assessing how groups of conditions may or may not relate to each other (in this case using Euclidean distance) based on the accumulated influence of each phenotype measured (in this case gene expression). Principal component analysis was carried out on the published muscle gene expression data using the 150 probe sets. In R, the following command was implemented: pcaData < -prcomp[t(data(,)], center = T, scale = T, and the first 2 principal components were plotted and genes selected that fulfilled 2 criteria: *1*) demonstration of distinct and measurable location in the principal component analysis plot and *2*) detectable by real-time qPCR in the cell culture system.

## RESULTS

**Figure 1.**
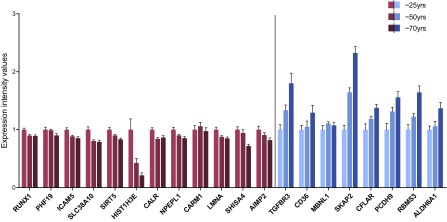
Expression intensity values for 20 age classifier genes (5) using previously published microarray data from human skeletal muscle across different age groups (4). Bars represent means ± sem, and data are normalized to average of young group. Red/blue bars represent down-/up-regulated genes with aging *in vivo.*

Twenty genes were selected after root–mean–square normalized principal component analysis of the original biologic age model that distinguished young from old healthy human skeletal muscle with ∼93% success (5). These 20 were chosen because they demonstrated a high degree of variance and because they were robustly expressed in both cell types used for the *in vitro* studies. We plotted the expression of these 20 genes in skeletal muscle profiles obtained from young (mean age 26 yr; *n* = 10), middle-aged (mean age 49 yr; *n* = 19), and old (mean age 70 yr; *n* = 16) healthy subjects using our previously published microarray data (4) to examine the changes across 4 decades (**Fig. 1**). This analysis demonstrated that there were several categories of patterns of gene expression change. Nine demonstrated a potentially linear pattern of change, while the remainder demonstrated a dramatic step either at young to middle age or middle age to old age. [This pattern is expected, as the selection process for the 150 genes relied on a nonlinear selection process (5).] As with the ontology/pathway analysis for the full 150 genes (5), this subset of 20 genes did not fall into any specific ontologic pathway. Furthermore, a manual search of the GenAge database revealed that only one of the 20 genes (*LMNA*) was found to be associated with aging in other model organisms. However, using a simple literature analysis, the known biologic functions attributed to the 20 genes was evaluated, with some biochemical or molecular properties relating to proposed mechanisms for aging (**Table 1**), including epigenetic regulation (*CARM1,* *HIST1H3E*), lipid metabolism (*CD36*), and stem cell function (*RUNX1*).

**TABLE 1.** Selected age classifier genes and their known biologic functions

No.	Gene symbol	Gene name	Direction with age	Biology
1	*RUNX1*	Runt-related transcription factor 1	Down	Transcription factor; differentiation of hemopoietic stem cells
2	*PHF19*	PHD finger protein 19	Down	Binds methylated histone H3 and recruits polycomb repressive complex 2
3	*ICAM5*	Intercellular adhesion molecule 5, telencephalon	Down	Transmembrane glycoprotein involved in adhesion
4	*SLC38A10*	Solute carrier family 38, member 10	Down	Putative neutral amino acid transporter
5	*SIRT5*	Sirtuin 5	Down	NAD-dependent protein deacetylase; mitochondrial
6	*HIST1H3E*	Histone cluster 1, H3e	Down	Replication-dependent histone; core component of nucleosome
7	*CALR*	Calreticulin	Down	Calcium binding protein in endoplasmic reticulum; protein folding; possible nuclear receptor modulation
8	*NPEPL1*	Aminopeptidase-like 1	Down	May catalyze removal of unsubstituted N-terminal AA from various peptides
9	*CARM1*	Coactivator-associated arginine methyltransferase 1	Down	Methylates histones and chromatin-associated proteins
10	*LMNA*	Lamin A/C	Down	Mutation linked to Hutchinson-Gilford progeria syndrome; nuclear membrane structural component; DNA replication; chromatin organization
11	*SHISA4*	Shisa homolog 4 (*Xenopus laevis*)	Down	Transmembrane protein; may inhibit Wnt and FGF signaling
12	*AIMP2*	Aminoacyl tRNA synthetase complex-interacting multifunctional protein 2	Down	Required for aminoacyl-tRNA synthase complex assembly; proapoptotic
13	*TGFBR3*	TGF, β receptor III (β-glycan, 300 kDa)	Up	Membrane proteoglycan that acts as coreceptor with other TGF-β receptor superfamily members
14	*CD36*	*CD36* molecule (thrombospondin receptor)	Up	Receptor for oxidized lipids
15	*MBNL1*	Muscle blind-like 1	Up	RNA binding; regulates splicing
16	*SKAP2*	Src kinase–associated phosphoprotein 2	Up	Adapter protein; actin assembly/stress fiber formation;
17	*CFLAR*	CASP8- and FADD-like apoptosis regulator	Up	Apoptosis regulator; lacks caspase activity; overexpression impacts on muscle satellite cell proliferation
18	*PCDH9*	Protocadherin 9	Up	Ca^2+^-dependent transmembrane protein important in cell adhesion in neural tissues
19	*RBMS3*	RNA binding motif, single-stranded interacting protein 3	Up	RNA binding protein
20	*ALDH6A1*	Aldehyde dehydrogenase 6 family, member A1	Up	Mitochondrial; valine and pyrimidine catabolic pathways

### siRNA-mediated knockdown of age classifier genes in human skeletal muscle and kidney cells

Ten genes, down-regulated *in vivo* with muscle age (Fig. 1), were selected for individual siRNA-mediated knockdown (2 independent siRNAs and a total of 60 independent cell cultures) to examine the interrelationship been each gene and the remaining 19 from the age classifier (5). Because the classifier was diagnostic for age across different tissue types, we wished to establish whether any individual gene knockdown mediated responses that were consistent across more than one human cell type. We chose to use human primary skeletal muscle cells because the age gene classifier was built on skeletal muscle tissue and renal epithelial cells, as the age genes were shown to be correlated with renal function over a 12-yr span (5). We verified that the siRNAs did not cause any obvious adverse effects related to cell health/viability using renal cells used for immunofluorescence staining against annexin V and propidium iodide to assess apoptosis status and evidence for necrosis, respectively (Supplemental Fig. 1). Initial real-time qPCR analysis revealed no effect of the transfection reagent on any of the genes analyzed (data not shown) in either cell type.

**Figure 2.**
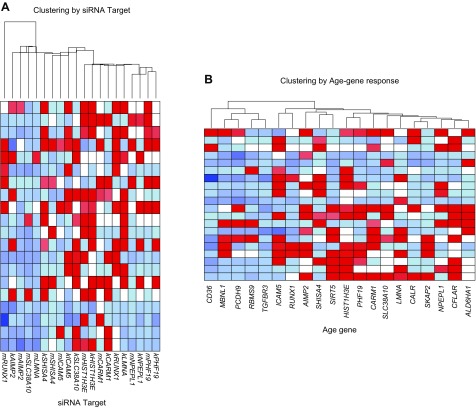
Heat maps of siRNA experiment data. Distance-based clustering (Euclidean) was used to determine global relationship across all siRNA experiments, grouping data set according to either siRNA target (*A*) or by age gene response (*B*). Each column represents mRNA expression data from single siRNA experiment, with every row being 1 of 20 measured genes. Letters “k” and “m” preceding each gene symbol represent data from kidney and muscle cells, respectively. Seventy percent of siRNA-targeted genes produced relatively consistent pattern of gene expression responses in both muscle and renal cells. Colors represent magnitude of change in expression for each gene (red, down-regulated; blue, up-regulated) with siRNA treatment *vs.* controls.

Individual knockdown of the 10 selected age classifier genes induced multiple expression changes in the remaining 19 genes. To determine the global relationship across all siRNA experiments, we used distance-based clustering (Euclidean) and grouped the entire data set according to siRNA target (**Fig. 2*A***) or according to gene (Fig. 2*B*). We observed that 70% of siRNA targeted genes produced a relatively consistent pattern of gene expression responses in muscle and renal cells (*i.e.,* the nearest neighbor of an siRNA in renal cells was the same siRNA in muscle cells) (Fig. 2*A*), while genes like *ICAM5* appeared to produce a more cell-type-specific response on the basis of this cluster analysis. Overall, the consistent response in both renal and muscle cells supports our original *in vivo* analysis (5).

Clustering by gene expression rather than by siRNA treatment revealed an intriguing result, where many of the genes (*CD36, MBNL1, PCDH9, RBMS9,* and *TGFBR3*) that are up-regulated *in vivo* with age in healthy muscle were up-regulated across all experiments (regardless of which gene was being down-regulated). This indicates that these genes may be up-regulated *in vivo* as a secondary response to down-regulation of key members of the 150 genes. We also noted, as expected, that genes down-regulated *in vivo* clustered together (Fig. 2*B*), partly reflecting the fact that we experimentally knocked down half of those genes while the remaining genes that were down-regulated *in vivo* nevertheless clustered with these down-regulated genes and not with the distinct group of *in vivo* up-regulated genes.

**Figure 3.**
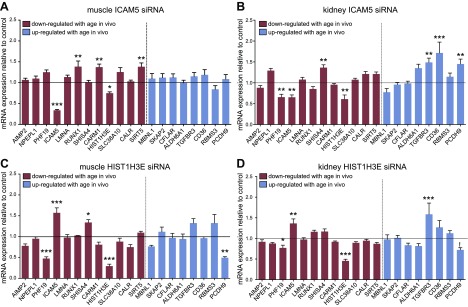
Impact of *ICAM5* and *HIST1H3E* knockdown on expression of age classifier genes in human skeletal muscle (*A*, *C*) and kidney (*B*, *D*) cells. Data are normalized to β-actin and expressed relative to controls (transfection reagent only). Bars represent means ± sem from 2 to 3 independent experiments and for 2 different siRNAs targeting *ICAM5* (*A*, *B*) or *HIST1H3E* (*C*, *D*) (*n* = 9–12 cell culture well replicates). Red/blue bars represent down-/up-regulated genes with aging *in vivo.* *P < 0.05, **P < 0.01, ***P < 0.001 *vs.* transfection reagent controls.

**Figure 4.**
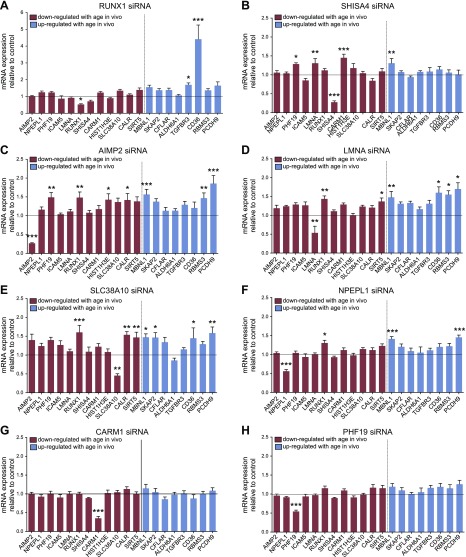
Impact of classifier gene knockdown on expression of age classifier genes in human skeletal muscle cells. Data are normalized to β-actin and expressed relative to controls (transfection reagent only). Each bar represents mean ± sem from 2 to 3 independent experiments and for 2 different siRNAs targeting *RUNX1* (*A*), *SHISA4* (*B*), *AIMP2* (*C*), *LMNA* (*D*), *SLC38A10* (*E*), *NPEPL1* (*F*), *CARM1* (*G*), or *PHF19* (*H*) (*n* = 9–12 cell culture well replicates). Red/blue bars represent down-/up-regulated genes with aging *in vivo.* *P < 0.05, **P < 0.01, ***P < 0.001 *vs.* transfection reagent controls.

**Figure 5.**
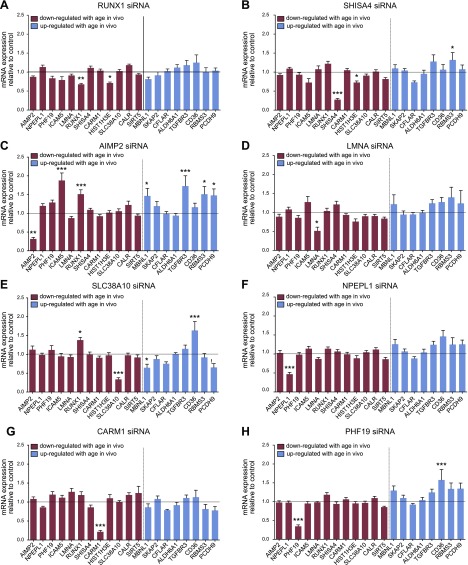
Impact of classifier gene knockdown on expression of age classifier genes in human kidney cells. Data are normalized to β-actin and expressed relative to controls (transfection reagent only). Bars represent means ± sem from 2 to 3 independent experiments and for 2 different siRNAs targeting *RUNX1* (*A*), *SHISA4* (*B*), *AIMP2* (*C*), *LMNA* (*D*), *SLC38A10* (*E*), *NPEPL1* (*F*), *CARM1* (*G*), or *PHF19* (*H*) (*n* = 9–12 cell culture well replicates). Red/blue bars represent down-/up-regulated genes with aging *in vivo.* *P < 0.05, **P < 0.01, ***P < 0.001 *vs.* transfection reagent controls.

There were some cell-type-specific patterns; for example, in human primary skeletal muscle cells, knockdown of *ICAM5, HIST1H3E, SHISA4, RUNX1 AIMP2*, *LMNA,* and *SLC38A10* had a significant impact on the other age genes overall (*P* < 0.05, *P* < 0.05, *P* < 0.05, *P* < 0.01, *P* < 0.01, *P* < 0.001, *P* < 0.001 *vs.* control, respectively; 2-way ANOVA main effect; **Fig. 3*A, C***), whereas siRNA-mediated knockdown of *NPEPL1, CARM1,* and *PHF19* mRNA had minimal impact on expression of the other age genes in these cells (**Fig. 4**). In human primary renal epithelial cells, *ICAM5, HIST1H3E,* and *AIMP2* had an overall effect on classifier gene expression (*P* < 0.001, *P* < 0.05, *P* < 0.001 *vs.* control, respectively; 2-way ANOVA main effect; Fig. 3*B, D*), whereas the remaining genes had little impact (**Fig. 5**).

In muscle cells, knockdown of *ICAM5* caused a significant change in expression of 4 of the other age genes (*HIST1H3E* was decreased and *RUNX1, CARM1,* and *SIRT5* were increased (Fig. 3*A*). *ICAM5* knockdown in kidney cells also down-regulated *HIST1H3E* (along with *PHF19*), while *SHISA4, TGFBR3, CD36,* and *PCDH9* were up-regulated (Fig. 3*B*). *HIST1H3E* knockdown also affected several age genes with a relatively consistent effect across muscle and renal cells (Fig. 3*C, D*). Thus, the response of *HIST1H3E* appeared consistent across cell types, and *HIST1H3E* was the most regulated gene *in vivo.* Knockdown of the remaining 8 genes had varying effects on expression of the other age classifier genes (Figs. 4 and 5). Overall, the degree of statistical significance for individual genes will be reflective of sample size, and in general, the more powerful methodology—cluster analysis—revealed coordinated gene expression changes.

### Effects of selected age-associated chemical mediators on expression of age classifier genes

Another key facet warranting exploration is the impact of putative positive/negative regulators of tissue aging on healthy tissue age genes. Thus, skeletal muscle cells were treated with selected age-related factors in an effort to assess whether each tissue age gene might be regulated by these aging-related chemicals. For each individual treatment, control genes known to be associated with that compound were measured as confirmation of activity in our cells (Supplemental Fig. 2). Initial experiments also verified that the vehicle controls DMSO and BSA had no effect on expression of the 20 age genes (Supplemental Fig. 3).

**Figure 6.**
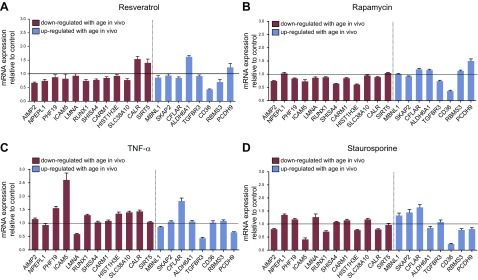
Impact of resveratrol (*A*), rapamycin (*B*), TNF-α (*C*), and staurosporine (*D*) treatment on expression of age classifier genes in skeletal muscle cells. Cells were treated for 24 h with 100 nM rapamycin, 50 μM resveratrol, 10 ng/ml TNF-α, or 10 nM staurosporine (*n* = 3), and experiments were performed over 3 passages. Data are normalized to β-actin and expressed relative to DMSO or BSA controls. Blue/red bars represent down-/up-regulated genes with aging *in vivo.*

Resveratrol is a naturally occurring polyphenolic compound with antioxidant activity that activates *SIRT1,* an NAD^+^-dependent histone deacetylase, and possibly *AMPK* (14, 15). Resveratrol has reported antiaging effects in yeast (14), flies (16), and, in some studies, mice (17, 18). Treatment of differentiated skeletal muscle cells with resveratrol had an overall effect on expression of the age classifier genes as assessed by ANOVA (*P* < 0.05; **Fig. 6*A***), but these changes were significantly different from the pattern of *in vivo* changes seen between old and young tissue (*P* = 0.0033 with Fisher’s exact test). Induction of *SIRT1* and *AMPK* gene expression by resveratrol confirmed expected features of resveratrol treatment (Supplemental Fig. 2).

Rapamycin is an antibiotic that binds to and inhibits mTOR, a nutrient-responsive kinase that influences metabolism and cellular growth *via* the PI3K/AKT pathway (19). Genetic or pharmacologic inhibition of mTOR extends the life span of *Caenorhabditis elegans* (20), *Saccharomyces cerevisiae* (21), and mice (18, 22). Rapamycin treatment reduced *EIF4E* and *S6K,* as expected (Supplemental Fig. 2). Rapamycin also had an effect on expression of the age classifier genes (*P* < 0.05 by 2-way ANOVA) and moreover induced a pattern of change that was not different from the pattern of changes noted *in vivo* with tissue age (Fig. 6*B**;* *P* = 0.2273 with Fisher’s exact test). Thus, rapamycin treatment altered gene expression in our cell studies in a manner similar to that observed with tissue age in healthy old muscle.

There are also compounds that actively regulate detrimental age-related phenotypes. It has been proposed that reduced autophagic capacity with aging may be responsible for increased expression of proinflammatory mediators that result from mitochondrial dysfunction and increased reactive oxygen species (ROS) production (23). TNF-α was also chosen as a proaging factor because aging in older humans has been correlated with a proinflammatory phenotype (24). Staurosporine is a potent inducer of oxidative stress, and excessive ROS can impair oxidative phosphorylation and promote accumulation of mitochondrial DNA mutations, leading to widespread cellular damage and numerous features of aging (25, 26). Muscle cells were treated for 24 h with TNF-α and staurosporine, and changes in expression of positive control (Supplemental Fig. 2) and age classifier genes were examined. There was a main effect of both TNF-α and staurosporine treatment on gene expression (both *P* < 0.05 *vs.* vehicle controls; Fig. 6*C, D*), but again these changes were significantly different from the pattern of changes observed *in vivo* with aging (*P* = 0.0004 for staurosporine, *P* < 0.0001 for TNF-α with Fisher’s exact test). Thus, only rapamycin demonstrated an *in vitro* response that was consistent with what was observed *in vivo.*

## DISCUSSION

Extensive studies in animal models and human tissues have been undertaken to gain insights into the process of aging and have identified many genes that show age-dependent changes in expression (4, 27, 28). Nonetheless, in most cases, each individual study identified a different set of genes or did not apply rigorous classification methods to verify consistency. Further, for all of the epidemiologically derived molecular signatures of aging, the identified patterns of gene expression incorporate the additional influences of clinical disease and/or drug treatment (6, 29) and thus do not represent aging *per se.* The present study was undertaken to examine the regulation of our recently identified gene expression signature derived from healthy older muscle tissue (5). This set of 150 genes was able to reproducibly classify young *vs.* older human muscle, brain, and skin tissue across 7 independent cohorts. However, pathway analysis showed no enrichment for any particular biologic process. Thus, analyses into the regulation of these genes could help our understanding of healthy aging processes in humans if we could regulate the signature in human cells with drugs or if one or more genes in the list regulated the other members of the signature.

Our first set of experiments aimed to address whether there were interactions between the age classifier genes that could be driving the changes in expression with aging. In doing so, we found that none of the individual age genes, when down-regulated using siRNA, exactly mimicked alterations in mRNA seen *in vivo,* indicating it to be unlikely that a single gene is driving our RNA signature. Nonetheless, when the entire pattern of *in vitro* gene expression was examined, those genes up-regulated *in vivo* clustered together as a distinct group in the *in vitro* data, indicating a coordinated response to siRNA-mediated loss of expression of genes seen to be down-regulated in healthier older tissue *in vivo.* Whereas some genes analyzed had little impact on expression of other age genes (*PHF19, CARM1, NPEPL1*), clear interactions between other genes were observed, motivating the need for future investigation of the entire 150-gene set to identify the nature of the gene networks comprising the healthy aging signature.

One gene that influenced several of the age genes was *HIST1H3E*. There is accumulating evidence that widespread epigenetic changes occur throughout life, including changes to histone posttranslational modifications and DNA methylation patterns (30–32). For instance, deletion of histone methylation complex components extends life span in worms and flies (33, 34). The *HIST1H3E* gene encodes the H3.1, a core component of the nucleosome. Knockdown of *HIST1H3E* induced similar changes in both kidney epithelial and skeletal muscle cells, causing a reciprocal increase in the expression of *ICAM5* and *SHISA4,* and down-regulation of *PHF19* and *PCDH9. PHF19* is a polycomb-encoding gene that functionally interacts with methylated histone H3 (35). *SHISA4, PCDH9,* and *ICAM5* all encode cell surface proteins, but their potential links to epigenetic processes remain unclear. We analyzed microarray expression patterns of additional histone encoding genes within the same histone cluster as *HIST1H3E* across age in a skeletal muscle data set (Supplemental Fig. 4) to determine whether this age-dependent change was unique to the *HIST1H3E* gene. Although some genes within the cluster were down-regulated with age, changes in *HIST1H3E* were the most marked.

*ICAM5* also regulated age gene expression, but it appeared to affect different genes in kidney and skeletal muscle cells. *ICAM5* knockdown caused a reduction in *HIST1H3E* expression in both cell types, further supporting a regulatory link between this adhesion molecule and histone expression. In skeletal muscle cells, *ICAM5* influenced expression of *RUNX1,* a transcription factor linked to histone deacetylases; *CARM1,* a methyl-transferase that can methylate histones; and *SIRT5,* a protein deacetylase. This indicates that *ICAM5* may have an important role regulating gene expression and histone modification processes. Thus, further work will be required to explore the potential role of *ICAM5* in relation to aging and the possible link between alterations in intercellular communication and epigenetic regulation, 2 key features of the aging process. Differing responses between human kidney and skeletal muscle cells could have been due to differences in cell type or due to other gene family members having dominant roles in that cell type; or because the kidney cells were in a proliferative state and muscle cells were differentiating. Thus, even though the genes within this RNA signature are able to accurately classify age of multiple human tissues *in vivo,* the relative functional importance of these genes could vary in different cell types.

Although *RUNX1* had little impact on overall expression of the age classifier genes, knockdown did cause a significant increase in *CD36* expression in muscle cells. *CD36* is a transmembrane glycoprotein that has previously been reported to increase with aging and may be important for clearance of circulating oxidized lipids (36). *CD36* may also contribute to accumulation of lipids within cardiac myocytes and hence cardiomyopathy in aged mice (37). *CD36* has been shown to contain RUNX-binding elements, and *RUNX3* can repress *CD36* expression in myeloid cells (38). Little is understood about the potential link of *RUNX1* to age-related pathways, although it is a genomic mediator of adaptation to exercise (39, 40) and thus may be an example of a key determinant of age–gene–environmental interactions. Whether increased *CD36* expression with aging is protective or damaging is unclear, but this change may be the result of reduced expression of RUNX transcription factors.

Lamins form a major component of the nuclear lamina, which is crucial for genomic stability because it provides a scaffold for chromatin and protein complexes (41). Mutation of the lamin genes cause accelerated aging syndromes such as Hutchinson-Gilford progeria syndrome. Thus, it represented an ideal target to study in relation to our age genes. It has been reported that normal aging is associated with accumulation of progerin (the aberrant prelamin A isoform), which may be linked to histone modification and DNA damage (42), a feature that accompanies aging. *LMNA* knockdown in kidney cells had little impact on age gene expression, but in muscle, there appeared to be a general increase in mRNA abundance. This was also observed for *AIMP2,* a gene required for assembly of the aminoacyl-tRNA synthase complex (43), and *SLC38A10,* a neutral amino acid transporter. These observations suggest that reduced expression of these genes may have influenced general mRNA translation, resulting in general accumulation of mRNA transcripts. It would seem critical that a future analysis examines the posttranscriptional regulation of these markers of healthy older tissue using, for example analysis of the mRNA engaged with the translational machinery.

Our second major aim was to determine links between positive/negative aging compounds and our age genes. Each of the 4 age-related compounds screened caused significant changes in gene expression for positive control genes as assessed by ANOVA (Supplemental Fig. 2) and when compared to vehicle control, but only rapamycin yielded gene expression changes in a pattern that was consistent with what we observed *in vivo* with age (Fig. 1) (5). Indeed, Fisher’s exact test revealed that the RNA profile of rapamycin was not significantly distinguishable from *in vivo* muscle aging (*i.e.,* genes that were up-regulated *in vivo* were more frequently up-regulated by rapamycin, and *vice versa*). Although the directional changes and consistency with the *in vivo* pattern were established using unbiased statistical analysis, the directional overlap was not 100%. However, because we were comparing gene expression changes in a primary cell model system measured over periods of days to *in vivo* changes with aging over decades, one might not expect complete concordance. The protein kinase mTOR responds to a variety of signals including growth factors, energy status, stress, and amino acids to regulate cell growth and metabolism (19). mTOR forms part of 2 distinct complexes, mTORC1 and mTORC2; mTORC1 is a critical regulator of protein synthesis and autophagy, whereas mTORC2 may have a predominant role in glucose metabolism and cell survival. Rapamycin has been shown to specifically target mTORC1, but chronic treatment can also inhibit mTORC2 (44). With a growing number of studies reporting beneficial effects of rapamycin on extension of life span and health span (18, 20, 22), this indicates that these gene expression markers for healthy tissue age warrant further detailed investigation in relation to their potential regulation by or link with mTOR.

The effect of resveratrol on expression of the healthy age genes was assessed. Previous studies on resveratrol demonstrated its ability to extend life span in *C. elegans, Drosophila melanogaster,* and *S. cerevisiae* (14, 16). It was reported that the beneficial effects of resveratrol may be mediated through activation of sirtuins, though resveratrol can also activate *AMPK* signaling (14, 15). In rodents, reduced risk of death due to high-calorie feeding was observed in mice with resveratrol treatment (17). In humans, resveratrol has some reported benefits on metabolic parameters (insulin sensitivity, fat metabolism), mimicking a caloric restriction phenotype (45). In this study, changes in expression of the age genes did not significantly replicate the directional changes with healthy aging *in vivo,* despite seeing an effect of rapamycin, which is also viewed as a calorie-restriction mimetic. This implies that the age genes were not directly regulated by *SIRT1*- or *AMPK*-activated pathways.

We also examined whether our healthy aging RNA signature might be linked to any inflammatory or oxidative stress–related pathways. Inflammation-induced tissue damage has been proposed to accumulate with aging, potentially as a result of reduced autophagic capacity or buildup of senescent cells (23, 24). Accumulation of ROS is thought to lead to widespread cellular damage with aging, although conflicting evidence regarding the role of ROS in aging has been reported (26, 46, 47). Expression of many of the age classifier genes responded to treatment with TNF-α and staurosporine; however, the changes were inconsistent with the *in vivo* directional age changes, and it was clear that both treatments caused cell stress responses that would have mostly likely influenced thousands of other genes. This suggests that *in vivo,* these age genes are not being influenced by inflammatory or oxidative stress–related pathways.

In summary, we have demonstrated *in vitro* coordination of gene expression within our *in vivo* validated tissue age signature, as well as potential epigenetic interactions. Further analysis of the interactions between the entire 150 genes and their potential link to mTOR signaling is merited.

## Supplementary Material

Supplemental Data

## Abbreviations

BSA: bovine serum albumin
mTOR: mechanistic target of rapamycin
qPCR: quantitative PCR
ROS: reactive oxygen species
siRNA: small interfering RNA
