# Supplementary material for: A reverse genetics cell-based evaluation of genes linked to healthy human tissue age
Source: FASEB J. 2016 Oct 3;31(1):96–108. doi: 10.1096/fj.201600296RRR (PMC5161526; doi:10.1096/fj.201600296RRR)
Supplement: Supplemental Data [file supp_31_1_96__index.html]

A reverse genetics cell-based evaluation of genes linked to healthy human tissue age — Supplemental Data 

# A reverse genetics cell-based evaluation of genes linked to healthy human tissue age

## Supplemental Data

- Supplemental Data
- Supplemental Data
- Supplemental Data
- Supplemental Data
