# Supplementary material for: A reverse genetics cell-based evaluation of genes linked to healthy human tissue age
Source: FASEB J. 2016 Oct 3;31(1):96–108. doi: 10.1096/fj.201600296RRR (PMC5161526; doi:10.1096/fj.201600296RRR)
Supplement: Supplemental Data [file supp_fj.201600296RRR_Supplemental_Figure1.docx]

**Supplementary Figure 1**


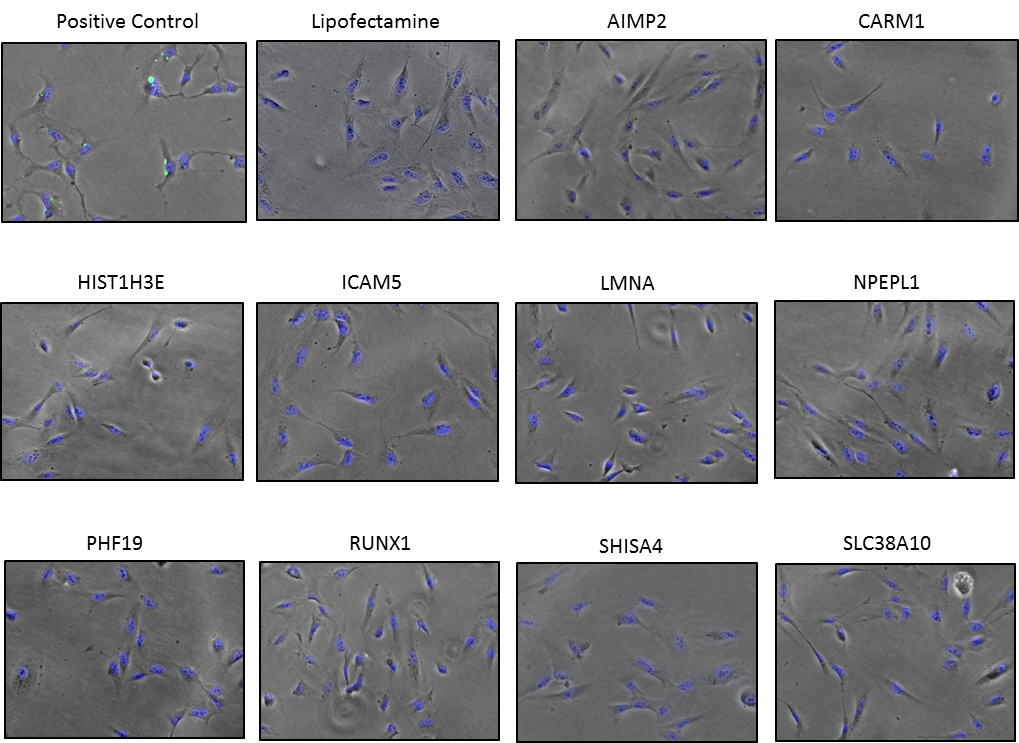


**Annexin V/DAPI staining of human renal epithelial cells following siRNA transfection**. Cells were grown to ~50% confluency and transfected for 48 h with siRNA targeting one of 10 age classifier genes. Cells were incubated with Annexin V-FITC and propidium iodide, before being fixed and stained with DAPI solution. Images of Annexin V staining are overlaid with DAPI and light microscope images.
