# Supplementary material for: A reverse genetics cell-based evaluation of genes linked to healthy human tissue age
Source: FASEB J. 2016 Oct 3;31(1):96–108. doi: 10.1096/fj.201600296RRR (PMC5161526; doi:10.1096/fj.201600296RRR)
Supplement: Supplemental Data [file supp_fj.201600296RRR_Supplemental_Figure2.docx]

**Supplementary Figure 2**

A

BSA

DMSO

TNF-α


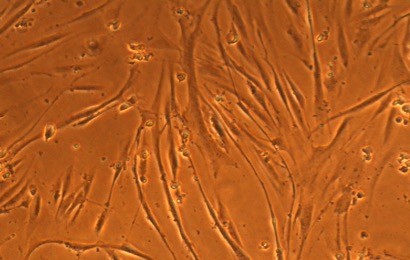

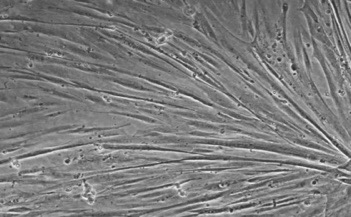

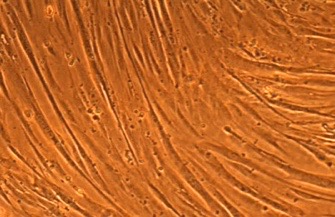

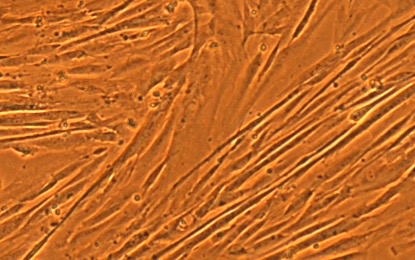

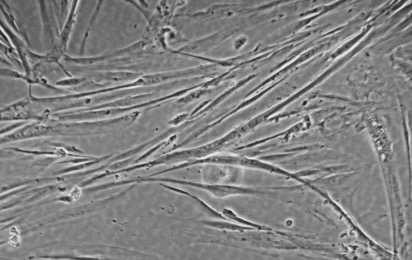

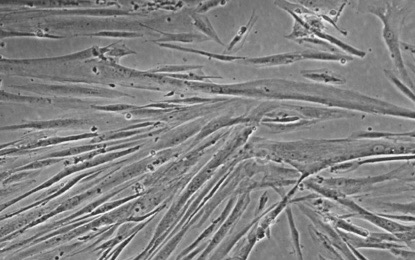


Resveratrol

Rapamycin

Staurosporine

B

**Images of skeletal muscle cells and expression of marker genes treated with rapamycin, resveratrol, TNF-α and staurosporine**. Cells were treated for 24 h with 10 ng/ml TNF-α, 100 nM rapamycin, 50 μM resveratrol or 10 nM staurosporine (n=3), and experiments were performed over three passages. Data are normalised to beta actin and expressed relative to controls (BSA for TNF-α, DMSO for rapamycin, resveratrol and staurosporine).
