# Supplementary material for: A reverse genetics cell-based evaluation of genes linked to healthy human tissue age
Source: FASEB J. 2016 Oct 3;31(1):96–108. doi: 10.1096/fj.201600296RRR (PMC5161526; doi:10.1096/fj.201600296RRR)
Supplement: Supplemental Data [file supp_fj.201600296RRR_Supplemental_Figure3.docx]

**Supplementary Figure 3**

**Impact of DMSO and BSA on expression of age classifier genes in skeletal muscle cells.** Cells were treated for 24 h with DMSO (0.3% v/v) and BSA (0.0001% w/v; n=3 for both treatments). Data are normalised to beta actin and expressed relative to untreated controls. Red/blue bars represent down/up-regulated genes with aging in vivo.
