# Supplementary material for: A reverse genetics cell-based evaluation of genes linked to healthy human tissue age
Source: FASEB J. 2016 Oct 3;31(1):96–108. doi: 10.1096/fj.201600296RRR (PMC5161526; doi:10.1096/fj.201600296RRR)
Supplement: Supplemental Data [file supp_fj.201600296RRR_Supplemental_Figure4.docx]

**Supplementary Figure 4**

**Expression intensity values for selected histone genes using microarray data from human skeletal muscle across different age groups (Phillips *et al*. 2013)**. Bars are mean ± SEM.
